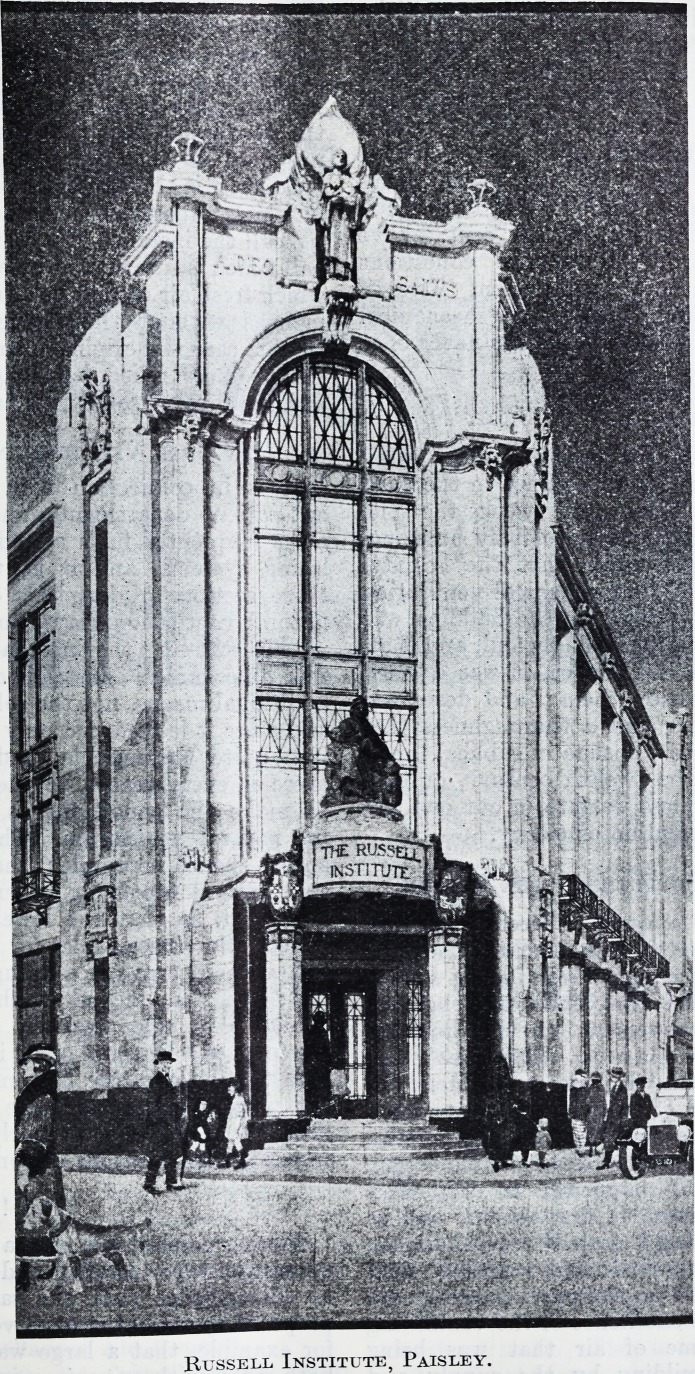# The Majesty of Motherhood

**Published:** 1924-10

**Authors:** 


### THE MAJESTY OF MOTHERHOOD.

### THE RUSSELL INSTITUTE AT PAISLEY.

By the courtesy of the Medical Officer of Health for Paisley, Dr. G. V. T. M'Michael, we are able to reproduce a drawing of the building which will be to the citizens of Paisley as a sign that the care of the mother and of the child is a worthy and a noble office. Too often the value and the nobility of the work of Maternity and Child Welfare is lost sight of because much of it is carried out in unpretentious and often mean buildings. One at least of Paisley's citizens, Miss Russell, of Muirfield, Paisley, recognising that the thing is worth doing, will see to it that it is done well. She has made to Paisley the very handsome offer of a Central Medical Institute for mothers and children in which all the numerous clinics for mothers and children will be assembled under one roof.

### A Handsome Building.

Miss Russell and her advisers have lost no time in pushing on with the scheme. They have acquired an ideal central site. Plans have been prepared, properties demolished to clear the site, and building operations begun. The plans show a handsome structure, consisting of three principal floors, a basement and an attic. The building will be of reinforced concrete and fireproof; the frontages to the streets will be built with dressed stone, with a base of black granite. The architects have consistently kept in mind the importance of securing the greatest possible amount of light, and the building has been planned accordingly.

Russell Institute, Paisley.

### Many Departments.

The first and second floors will be devoted to the various clinics for the inspection and treatment of school children, together with the administrative offices of the School Medical Department. To the left of the entrance is the Tuberculosis Department, consisting of a large waiting room, two dressing cubicles, and consulting room with a small dark room attached for laryngoscopic work. Across the passage from the Tuberculosis Department is the Clinic for dealing with verminous children; in one room the children discard their infested clothing, pass through to have a bath, and then into another dressing room, where they receive their clean clothing, which has meantime been passed through a disinfecting chamber. To the right of the entrance is the Maternity and Child Welfare Department, consisting of a spacious waiting room, with nurse's office, two dressing cubicles, medical officer's consulting room, and a treatment room for minor ailments, etc.; all the various rooms communicate direct with each other. The dispensary and various stores, etc., are also situated on the ground floor.

On the first floor is the Minor Ailment Clinic, the Special Treatment Clinic, for dealing with diseases of the eye, ear, nose, throat, etc., the accommodation consisting of waiting room, operating theatre and treatment room, recovery room, dark room, etc. On this floor also is the main office of the School Medical Department.

## Figures and Tables

**Figure f1:**